# Novel mutations in *CRB1* gene identified in a chinese pedigree with retinitis pigmentosa by targeted capture and next generation sequencing

**DOI:** 10.18632/oncotarget.12971

**Published:** 2016-10-28

**Authors:** Lan Lu, Xizhen Wang, David Lo, Jingning Weng, xiaohong Liu, Juhua Yang, Fen He, Yun Wang, Xuyang Liu

**Affiliations:** ^1^ Department of Ophthalmology, Fujian Medical University Union Hospital, Fuzhou, Fujian, 350001, China; ^2^ Shenzhen Key Laboratory of Ophthalmology, Shenzhen Eye Hospital, Jinan University, Shenzhen, Guangdong, 518000, China; ^3^ Department of Internal Medicine, Danbury, CT 06810, USA; ^4^ Department of Ophthalmology, The People's Hospital of Baoan Shenzhen, Guangdong, 518101, China; ^5^ Biomedical Engineering Center, Fujian Medical University, Fuzhou, Fujian, 350001, China

**Keywords:** retinitis pigmentosa, pedigree, next generation sequencing, mutation

## Abstract

**PURPOSE:**

To detect the disease-causing gene in a Chinese pedigree with autosomal-recessive retinitis pigmentosa (ARRP).

**METHODS:**

All subjects in this family underwent a complete ophthalmic examination. Targeted-capture next generation sequencing (NGS) was performed on the proband to detect variants. All variants were verified in the remaining family members by PCR amplification and Sanger sequencing.

**RESULTS:**

All the affected subjects in this pedigree were diagnosed with retinitis pigmentosa (RP). The compound heterozygous c.138delA (p.Asp47IlefsX24) and c.1841G>T (p.Gly614Val) mutations in the Crumbs homolog 1 (*CRB1*) gene were identified in all the affected patients but not in the unaffected individuals in this family. These mutations were inherited from their parents, respectively.

**CONCLUSION:**

The novel compound heterozygous mutations in *CRB1* were identified in a Chinese pedigree with ARRP using targeted-capture next generation sequencing. After evaluating the significant heredity and impaired protein function, the compound heterozygous c.138delA (p.Asp47IlefsX24) and c.1841G>T (p.Gly614Val) mutations are the causal genes of early onset ARRP in this pedigree. To the best of our knowledge, there is no previous report regarding the compound mutations.

## INTRODUCTION

Retinitis pigmentosa (RP) is a hereditary neurodegenerative retinal disease characterized by progressive loss of photoreceptors. The ocular phenotype of this disease include pigmentary retinopathy, arteriolar narrowing, waxy pallor of the optic disc, cystic macular lesions, cataracts and refractive errors [1–5]. Patients usually present with decreased night vision and loss of peripheral vision. The diverse pathogenic genes result in varied clinical manifestations. For example, previous studies showed that the severity of refractive errors in patients with RP was associated with the location of the mutated gene and/or the type of genes involved [5–7].

There are no known risk factors for RP except genetic predisposition. Approximately 70% of patients have a positive family history [8]. In the Netherlands, van den Born et al. estimated that ~63% of patients with RP had a positive family history, with ~30% inherited in an autosomal-recessive pattern, 22% autosomal-dominant, 10% X-linked and the remaining 37% isolated cases [9]. To date, more than 100 gene loci related to RP have been identified [10] and at least 17 genes are believed to be involved in ARRP. Gene mutations have been found in ~50% of ARRP cases [11]. The common known types of mutations causing ARRP in Israeli and Palestinian populations are located in *CRB1* [12]. Corton M et al reported that *CRB1* mutations frequently caused early-onset retinal dystrophies in Spanish populations [13]. Recently, approximately 194 mutations related to the pathogenesis of ARRP were identified in *CRB1* [14].

Previous studies demonstrated that targeted-capture NGS can precisely and rapidly identify genetic defects [15–16]. Several novel mutative genes have been identified via the NGS approach in patients with RP [17]. Booij et al analyzed a group of 35 unrelated patients with autosomal recessive juvenile retinitis pigmentosa via NGS and found that 12 patients had mutations. These mutations occurred in *CRB1* (11%), GUCY2D (11%), RPE65 (6%), and RPGRIP1 (6%) [11]. Yang et al reported that mutations in *CRB1* were found in four Chinese families as well as in some sporadic subjects with RP, with a 5.8% gene mutation frequency in *CRB1* [14].

In this study, a Chinese pedigree with ARRP was studied and the compound heterozygous mutations in *CRB1* were identified using NGS. To the best of our knowledge, these mutations in *CRB1* have not been reported previously.

## RESULTS

### Clinical findings

**Figure 1:**
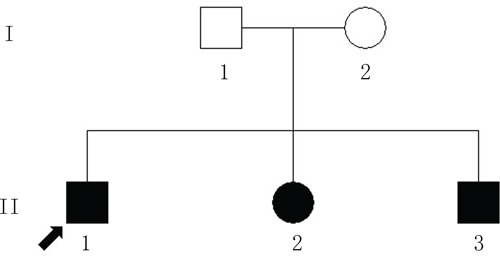
The Chinese pedigree with autosomal-recessive retinitis pigmentosa (ARRP) The circles indicate females; the squares indicate males. The filled shapes indicate the affected individuals with RP. The arrow signifies the proband.

The proband (Figure 1 = patient II:1, Figure 2A) is a 14-year-old male. Onset of disease was at age 2. He presented with diminished night vision and subsequent progressive loss of his peripheral and central vision, as well as reduced color vision and night blindness. On his most recent visit, his best corrected visual acuity (BCVA) was CF/30cm (+7.00DS+2.00DDC×105°) in the right eye and 0.03 (+9.00DS+1.50DDC×77°) in the left eye. The intraocular pressures were normal bilaterally. There was 15° esotropia in the left eye. Bilateral fundus examination showed a waxy, pale-appearing optic disc with markedly attenuated retinal arterioles. There were extensive typical bone spicules and round pigment clumping. OCT showed that marked atrophy changes in the macular region (Figure 3). The proband's brother (Figure 1= patient II:2, Figure 2B) was a 9-year-old male. Disease onset was also at age 2, presenting with similar symptoms and clinical signs as his proband brother. On his most recent visit, the BCVA was 0.2 (+7.75DS/+1.00DDC×100°) in the right eye and 0.1 (+8.50DS/+1.00DC×80°) in the left eye. Fundus patterns and OCT imaging were similar to his proband brother. The disease of the proband's sister (Figure 1 = patient II:3, Figure 2C) was a 7-year-old female. Disease also occurred at the age of 2, presenting with poor night and central vision. On her most recent visit, her best-corrected visual acuity was 0.3 in both eyes. Optometry values were as follows: 0.3 (+9.75DS/+2.25DC × 75°) right eye and 0.3(+10.50DS/+ 2.75DC × 95°) left eye. Fundus examination showed normal optic discs and attenuated retinal arterioles in both eyes. There was an area of hypo-pigmentation with localized bone–spicule pigmentation in the macular region. ERG responses were extinguished. Rod ERG b-wave disappeared in each eye. (Figure 4)

**Figure 2:**
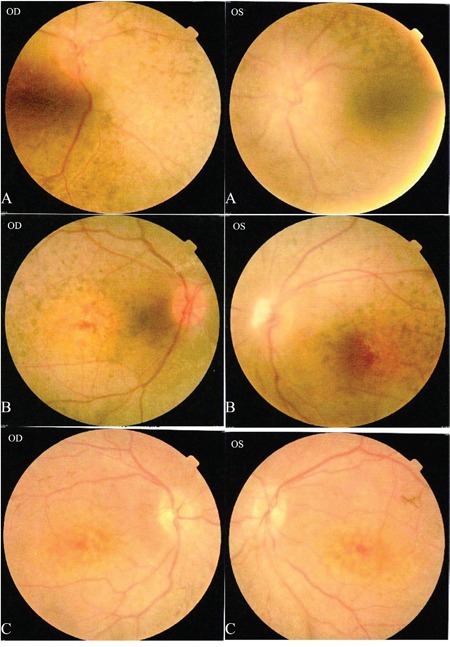
Patients II: A: II:1, B: II:2, C: II:3 Fundus photography in both eyes for patients II:1-3: **A.** and **B.** show a waxy pale-appearing optic disc, attenuated retinal arterioles and RPE atrophy. **C.** shows normal optic discs and attenuated retinal arterioles in each eye. There is an area of hypo-pigmentation with localized bone–spicule pigmentation in the macular region.

**Figure 3:**
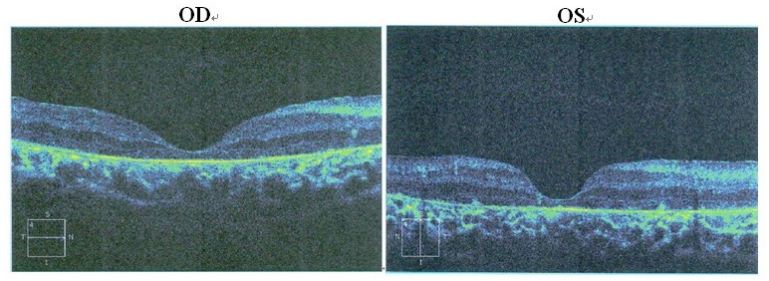
Patient II:1. OCT of the retinal pigment epithelium OCT shows marked atrophy of the retinal pigment epithelium and loss of photoreceptors. Cystic cavities within the inner and outer nuclear layers are noticed in both eyes. The retinal nerve fiber layer in the macular region is thick, with preservation of the foveal architecture.

**Figure 4:**
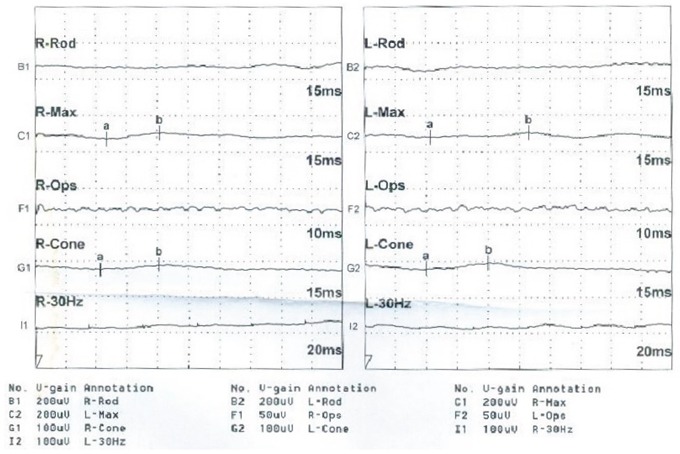
Patient II:3 ERG responses are extinguished, as interpreted by the reduction in amplitude and b-wave disappearance in each eye.

### Genetic findings

**Figure 5:**
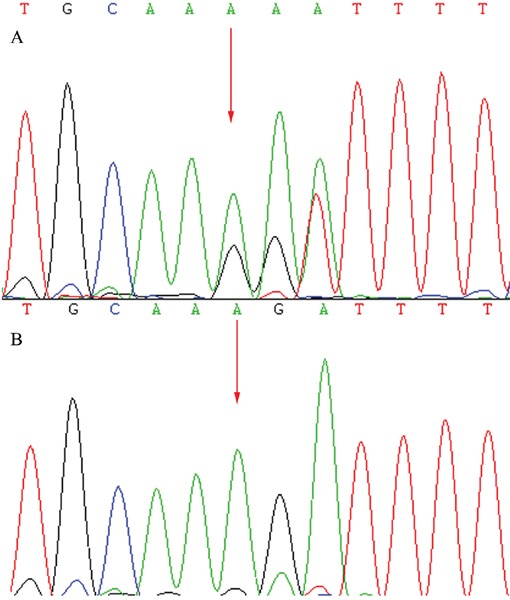
Sequencing results of the *CRB1* gene: Sequence analysis showed a heterozygous mutation c.19Y4NSiG8kkzwWjMD17euEaQ5PErpwxWkP (Red arrow indicates the location of the mutation) **A.** Patients. **B.** Unaffected mother.

**Figure 6:**
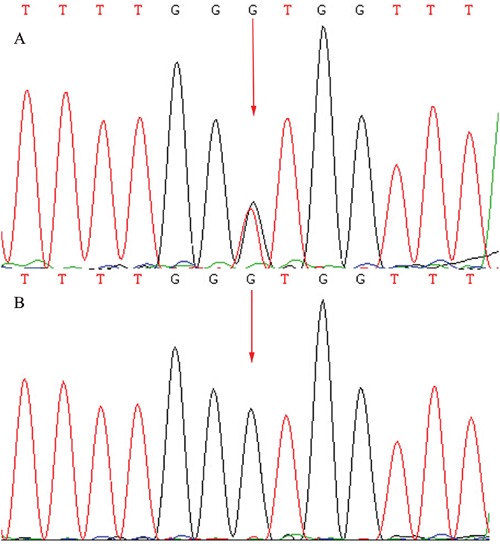
Sequencing results of the *CRB1* gene: Sequence analysis showed a heterozygous mutation c.1841G>T (p.Gly614Val) transversion in exon 6 (Red arrow indicates the location of the mutation) **A.** Patients. **B.** Unaffected father.

A total of 57 variants of candidate genes related to ARRP, including *RP1*, *RP2*, *RPGR*, *RHO*, *PRPH2*, and *CRB1*, were detected in the proband. The compound mutations, c.138delA and c.1841G>T, in *CRB1* were identified in the proband using gene chip sequencing. This compound heterozygote for c.138delA (p.Asp47IlefsX24) and c.1841G>T (p.Gly614Val) mutations (Figures 5 and 6) were verified in his affected siblings. The unaffected father is a heterozygote carrier of c.1841G>T (p.Gly614Val), and the unaffected mother is a heterozygote carrier of c.138delA (p.Asp47IlefsX24). There were no other candidate gene mutations found in this pedigree.

## DISCUSSION

*CRB1* mutations are associated with a series of autosomal recessive retinal dysthrophies such as Leber congenital amaurosis (LCA), [17–19] early onset RP, [20–21] and preserved para-arteriolar retinal pigment epithelium [18]. RP is one of the most common types of retinal dystrophy in China with a prevalence of nearly 1:4000 [19]. RP may occur as an isolated sporadic disorder, or inherited as an autosomal-dominant, autosomal-recessive, or X-linked pattern. The genes most frequently linked with RP include *RHO*, *USH2A*, *RPGR*, *CRB1*, *GUCY2D*, and *RPGPIP1* [11]. Previous studies reported *CRB1* mutations in ARRP in diverse populations [2, 11, 12, 14]. This study identified a pair of novel compound mutations in *CRB1* in a Chinese pedigree with ARRP. All affected patients in the second generation had compound heterozygous mutations for c.138delA (p.Asp47IlefsX24) and c.1841G>T (p.Gly614Val) in *CRB1*.

C*RB1* consists of 12 exons and 11 introns. It encodes two proteins of 1376 and 1406 amino acids and functions as an extra-cellular protein with a signal peptide. This protein contains 19 epidermal growth factor (EGF)-like domains, 3 laminin A globular (AG)-like domains, and C-type lectin (CTL), It plays an important role in the development of the retina [23–25]. Mutations in *CRB1* may restrain retinal development and result in the loss of photoreceptor signaling. Yang et al. reported patients with c.3460T>A and c.4207G>C mutations in *CRB1* presented with night blindness at the age of 20 and progressive vision lose [14]. In the present study, patients with compound c.138delA and c.1841G>T mutations in *CRB1* presented with visual impairment accompanied with high hyperopia at the age of 2. The parents, who carried either c.138delA (p.Asp47IlefsX24) or c.1841G>T (p.Gly614Val)) mutation, were spared from RP, while the three offspring who carried both two mutations from their parents presented with typical RP. The deletion mutation c.138delA results in truncated proteins with the absence of *CRB1* products such as transmembrane and cytoplasmic domains. This may stop protein translation. The c.1841G>T mutation caused a replacement of glycine (Gly) with Valine (Val) at the codon 614 which is localized on the 12th EGF-like domain, a highly conserved region. These compound mutations, therefore, seems to be responsible for the pathogenesis of the retinal degeneration seen in this family.

We are aware that all affected subjects in the present study had high hyperopia with spherical equivalent (SE) more than +7.00 diopters (D). Sieving et al analyzed 268 eyes with RP and found a mean SE was at −1.86 diopters [5]. Thomas et al reported that mutations in RP1 in patients with ARRP had SE median at −4.0 diopters, while without RP1 mutation had SE median less than −1.0 diopters [6]. Hanein et al showed that mutations in GUCY2D, RPGRIP1, CRX or CEP290 in patients with Leber congenital amaurosis frequently had high hyperopia [7]. Those studies imply that the types and degrees of refractive errors may be correlated with the types of mutations involved, patients with hyperopic refractive errors may predominantly involve *CRB1*, *GUCY2D*, *RPGRIP1*, *CRX* or *CEP290* mutations in patients with inherited retinal dystrophies.

## MATERIALS AND METHODS

### Subjects

All three patients in the second generation and two unaffected parents were enrolled in this study. No consanguineous marriage was noticed in the family. This study was approved by the Medical Ethics Committee of the Shenzhen Eye Hospital, Jinan University. Prior to participating in this study, all subjects were given a detailed explanation of the study. Informed consent was obtained from all participants according to the principles of the Declaration of Helsinki.

### Clinical examination

All study subjects in this family underwent a complete ophthalmic examination, including visual acuity test with linear Snellen, best corrected visual acuity, intraocular pressure (IOP) measurement, slit-lamp biomicroscopy, and funduscopic examination. Other examinations included fundus photography, visual field testing, full-field electroretinography (ffERG, Diagnosis LLC, Lowell, United States) recorded in accordance with the guidelines of the International Society for Clinical Electrophysiology of Vision (ISCEV), [15] and optical coherence tomography (Spectralis System, Heidelberg Engineering, Heidelberg, Germany, SD-OCT). OCT parameters were used to analyze the retinal pigment epithelium and the retinal nerve fiber layer thickness in the retina.

### Criteria for the diagnosis of retinitis pigmentosa

Subjects with typical symptoms, including decreased night vision or night blindness, waxy optic disc pallor, narrowing arterioles, pigment deposits, and ERG amplitude reduction [16].

### Mutation screening and sequence analysis

Peripheral venous blood was collected from all study subjects. Genomic DNA was extracted from 200 μL peripheral venous blood using a QIAmp Blood DNA Mini Kit (Qiagen, Hilden, Germany) according to the manufacturer protocols. The integrity of the DNA samples was verified by 1% agarose gel electrophoresis. Fifty-seven candidate genes of non-syndromic inherited RP including the *CRB1* gene were chosen as candidate genes to be enriched in the designed sequencing panel using custom-designed NimbleGen SeqCap probe hybridization (Roche NimbleGen, Inc., Madison, WI, USA). Target-Capture sequencing of all coding exons and 20bp of their flanking intronic regions of these genes were performed on the proband to detect the possible disease-causing gene mutations. The indicated DNA samples with equal molar ratios were put into each flow cell along the displayed lanes. Polymerase Chain Reaction (PCR) was observed with HiSeq2000 (Illumina, Inc., San Diego, CA, USA) using the Sequencing-By-Synthesis (SBS) method. The data was analyzed for gene alignment with CASAVA v1.7 (Illumina Inc.) and NextGene (SoftGenetics, State College, PA, USA) software.

Sanger sequencing was used to confirm the indicated mutations and determine whether any of the remaining variants co-segregated with the disease phenotype in this family. PCR amplification was performed. PCR primers were designed by the Primer Premier 5 software. Components of 30 μl of PCR reaction mixture included 15 μl 2× Taq PCR Master Mix (SinoBio, Shanghai, China), 30 ng DNA, 1.0 μM of both forward and reverse primers, and 12 μl ddH_2_O. The PCR reactions were incubated at 94°C for 3 min, followed by 35 cycles of 94°C for 20 s, annealing for 30 s, and 72°C for 60 s, with final extension at 72°C for 5 min. Purification of PCR products were sequenced using an ABI 377XL automated DNA sequencer (Applied Biosystems, Foster City, CA). The assembly of DNA sequences followed the DNAStar (Madison, WI) software and compared pairwise with online Human Genome databases. All mutations and its variant were interpreted and classified based on the nomenclature recommended by the Human Genomic Variation Society (HGVS).

## CONCLUSION

The novel compound heterozygous mutations for c.138delA (p.Asp47IlefsX24) and c.1841G>T (p.Gly614Val) in *CRB1* were identified as the pathogenic gene for ARRP in a Chinese pedigree using NGS. To the best of our knowledge, this is the first report that these mutations in *CRB1* are responsible for the pathogenesis of ARRP.
